# Photoinduced Disaggregation of TiO_2_ Nanoparticles Enables Transdermal Penetration

**DOI:** 10.1371/journal.pone.0048719

**Published:** 2012-11-14

**Authors:** Samuel W. Bennett, Dongxu Zhou, Randall Mielke, Arturo A. Keller

**Affiliations:** University of California Center on the Environmental Implications of Nanotechnology and Bren School of Environmental Science and Management, University of California Santa Barbara, Santa Barbara, California, United States of America; RMIT University, Australia

## Abstract

Under many aqueous conditions, metal oxide nanoparticles attract other nanoparticles and grow into fractal aggregates as the result of a balance between electrostatic and Van Der Waals interactions. Although particle coagulation has been studied for over a century, the effect of light on the state of aggregation is not well understood. Since nanoparticle mobility and toxicity have been shown to be a function of aggregate size, and generally increase as size decreases, photo-induced disaggregation may have significant effects. We show that ambient light and other light sources can partially disaggregate nanoparticles from the aggregates and increase the dermal transport of nanoparticles, such that small nanoparticle clusters can readily diffuse into and through the dermal profile, likely via the interstitial spaces. The discovery of photoinduced disaggregation presents a new phenomenon that has not been previously reported or considered in coagulation theory or transdermal toxicological paradigms. Our results show that after just a few minutes of light, the hydrodynamic diameter of TiO_2_ aggregates is reduced from ∼280 nm to ∼230 nm. We exposed pigskin to the nanoparticle suspension and found 200 mg kg^−1^ of TiO_2_ for skin that was exposed to nanoparticles in the presence of natural sunlight and only 75 mg kg^−1^ for skin exposed to dark conditions, indicating the influence of light on NP penetration. These results suggest that photoinduced disaggregation may have important health implications.

## Introduction

Nanoparticles (NPs) are used increasingly in many industrial, commercial and personal care products to replace bulk size materials [1], [2]. Based largely on scale, NPs exhibit unique physicochemical properties that require a better understanding of the biological and environmental behavior and implications [3], [4]. Preliminary research suggests that NPs may be more reactive and toxic than their bulk sized counterparts [4]. The bioavailability and toxicity of NPs in environmental and biological systems is influenced by the degree of particle aggregation, with smaller more dispersed particle generally more bioavailable and toxic [4]–[6]. Bare NPs without a stabilizing coating or cap will rapidly aggregate in most aqueous systems to well over 100 nm [7]–[11]. Classical colloid theory has been the basis for predicting the balance of forces that control NP aggregation, including van der Waals; electrostatic and acid base interactions, as well as steric repulsion and hydrophobic hindrance [8]–[11]. Our results indicate that a key phenomenon has been overlooked by previous research, namely the effect of sunlight, a common environmental condition, on NP aggregation state. Since NP size and degree of aggregation are critical properties in numerous applications, e.g., material synthesis, biomedical imaging; food-product coloration and stabilization; paint stabilization; sunscreen and cosmetics; and environmental remediation, fate and transport, this phenomenon can be important and useful for many disciplines [3], [12]. Although sunlight may also have an effect on larger colloids, it is likely to be minimal; it is most relevant at the nanoscale.

Much of the preliminary work associated with the environmental implications of NPs has pointed to the importance of NP aggregation, where the degree of aggregation can serve to estimate key environmental and ecologically important processes, e.g., transport, photoactivity, and bioavailability [8], [13]–[18]. NP transport in environmental media is a strong function of aggregate size, which influences their Brownian motion, sedimentation, deposition, filtration and straining [3], [11]–[18]. Animal toxicity studies have also shown a relationship between particle size and toxicological outcomes, where smaller particles can more readily transport *in vivo* and may lead to increased toxicity [19]–[21].

Since TiO_2_ photocatalytic reactions can produce free radicals, there have been a number of studies that investigated whether TiO_2_ can penetrate human skin [19]–[20], [22]. In an analysis of four sunscreen formulations, researchers found that the particle size of the raw materials were not changed, *i.e.*, the initial nanoparticle materials remained as nanoparticles in the sunscreen formulations [23]. Most investigations have shown that TiO_2_ NPs remain in the stratum corneum, the outermost layer, although some researchers have shown TiO_2_ NPs can penetrate deeper via the hair follicles [19]–[20], [22]. Although smaller particles have a greater ability to transport *in vivo*, convincing evidence that TiO_2_ NPs transport past or through the stratum corneum is lacking [20]. Tinkle et al [2003] have shown size dependent penetration of 0.5, 1, 2 and 4 µm BeO spherical particles through the stratum corneum into the epidermis of flexed human skin [21]. Wu and coworkers [2009] exposed TiO_2_ NPs, with sizes ranging from 4 to 60 nm, *in vivo* and *in vitro* to porcine skin and to hairless mice, and found that TiO_2_ did not diffuse beyond the stratum corneum in the isolated porcine skin [20]. However, after sub chronic dermal exposure of TiO_2_ NPs to *live* pig ears, particles were able to cross the stratum corneum, with transport dependent on particle size, with the smallest particles penetrating into the deepest layer of the epidermis [20]. After a 60-day dermal exposure of either 4, 10, 25 or 60 nm TiO_2_ particles to hairless mice, TiO_2_ was found in the skin, subcutaneous muscle, liver, heart, lungs, spleen and brain. The distribution of particles in the body was strongly dependent on particle size, where the smaller particles distributed more widely and had much greater toxicity, resulting in reduced body weight, keratinized skin, thinner dermis, focal necrosis in the liver and minor lung lesions; no pathologies were associated with bulk TiO_2_ [20]. Although Wu and coworkers showed TiO_2_ transport through the stratum corneum in *live* pigs, similar to other researchers, they did not observe NP penetration into the deeper layers of the isolated skin sections [19]–[22], [24].

In this work, we demonstrate that the absorption of light provides enough energy to partially disaggregate TiO_2_ NPs in aqueous media, releasing small particles from the larger aggregate. We find that light provides NPs with enough energy to shift the secondary minimum and subsequently release a few particles from the larger aggregate. Our work shows that photoinduced disaggregation has the potential to increase NP transport *in vivo* and possibly within the environment. We used porcine skin as a model biological tissue and show that sunlight facilitates NP penetration into the viable layers of the skin.

## Materials and Methods

### Materials

P25 TiO_2_ was obtained from Evonik Degussa Corporation (USA). The primary particle diameter was 27 (±4) nm, as measured by transmission electron microscopy (TEM) (Figure S1). Porcine skin was obtained from a local abattoir (Albertson's, Inc and used with permission). Adipose and connective tissues were removed from the skin by blunt dissection. Filters (1000 K and 100 K Microsep) with 100 and 10 nm nominal pore sizes were obtained from Pall Life Sciences (Pall Corporation, USA).

### Irradiation Experiments

**Figure 1:**
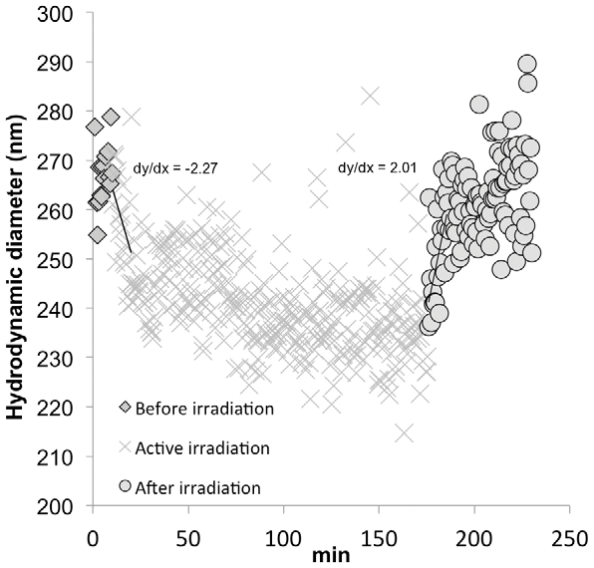
Real-time TiO_2_ NP disaggregation and re-aggregation in deionized water with a Xe lamp. The reaction was isothermal at 24.1°C. The ⧫ series represent size measurements of the dispersion for 10 minutes before light exposure. The **x** series represent size measurements during exposure to the full spectrum of the Xe lamp. The ○ series represent size measurements at the conclusion of light exposure. The rates of disaggregation and aggregation are also shown.

Light exposures were either conducted with natural sunlight (Santa Barbara, CA, 34.4125°N, 119.8481°W) or a 75 W xenon-arc lamp (Optical Building Blocks, Inc., USA) powered by a regulated power supply ( Xe spectrum in Figure S2). The spectrum and absolute irradiance of the Xe lamp were measured using a spectrometer (USB 4000, Ocean Optics, USA) with a calibrated cosine corrector that allows light collection from a 180° field of view. A power meter (842-PE, Newport Portable Optical Power Meter) outfitted with a silicon diode sensor was used as an additional instrumental method to verify light intensity. The total irradiance of the 75 W Xe arc lamp was 300 W m^−2^. Mid-day sunlight intensities in Santa Barbara, CA, range between 500 and 800 W m^−2^ [25], KG1, UG3 and 1000 nm bandpass filters were obtained from Schott Glass (USA) and used to filter the Xe light in specific experiments. Size and ζ-potential were measured with a Malvern Zetasizer Nano-ZS90 (Malvern, Inc., UK), except the real-time measurements (Figure 1), which were performed using Dynamic Light Scattering (DLS) with a BI-200SM (Brookhaven Instruments, USA). Sonication was done with a Sonicator 4000 (Misonix Ultrasonic, USA) fitted with a microtip.

### Photoinduced disaggregation

To prepare the NP stock suspension, the NPs were weighed, placed in NanoPure water (NanoPure Diamond, Barnstead, MA) and sonicated (Misonix Ultrasonic, USA) for 1.5 minutes, with ca. 10 W. To prepare the initial 100 mg l^−1^ NP dispersions for experimental disaggregation trials, 100 µl of 1000 mg l^−1^ stock were diluted in 900 µl of unbuffered deionized water (DI). The pH of the unbuffered solutions was generally 5.5 (±0.3). The 1000 µl samples for disaggregation were placed into 1 cm plastic cuvettes. The samples were not stirred, and to ensure settling of NPs during exposure did not influence the DLS size measurements several trials were well mixed after exposure and then measured. Immediately after light exposure, 1000 µl samples of the NP dispersions were placed in the Zetasizer for size and ζ-potential measurements; the samples were measured within one minute. For the real-time measurements reflected in Figure 1, 1000 µl samples were dispensed in a glass cuvette and placed inside the Brookhaven DLS that is open to the air and allows laser measurement through the side of the cuvette with simultaneous irradiation by the Xenon lamp from 20 cm above the sample. Real-time measurements were kept isothermal at 24.1°C with a recirculating water bath. The sunlight and xenon lamp exposed samples were measured at 25°C, although the sunlight irradiated samples heated to ca. 30°C after exposure.

For the experiments using the 100 nm pore filters, a 200 µL dose of 1000 mg L^−1^ TiO_2_ was dispensed directly onto the filter paper inside the receptor vial. The xenon lamp was positioned 25 cm above the filter. The permeate was acid digested, with four parts permeate added to 6 parts 60% concentrated H_2_SO_4_ and 40% saturated ammonium sulfate solution. The final permeate-acid solution was heated at 90°C for 1.5 hours, diluted 10 times with Nanopure water and then analyzed by inductively coupled plasma (ICP) using a Thermo iCap 6300 (Thermo Scientific, USA). NIST traceable titanium standards were obtained from High Purity Standards (South Carolina, USA) and used to produce a calibration curve ranging from 1 part per billion to 10 parts per million Ti. All disaggregation experiments were conducted at least ten times, except the Pall filter experiments which were conducted in triplicate.

### DLVO and force-energy calculations

The attraction force at the secondary minimum was obtained by evaluating the force-separation distance profile, constructed by summing the attractive van der Waals force and the repulsive electrostatic force based on DLVO theory, the parameters used can be seen in Table 1 (see also Supporting Information S1 for more details on DLVO).

**Table 1:** Parameters used in the DLVO calculation.

Parameters	TiO_2_
Hamaker Constant (J)	9.10×10^−20^ a
Zeta-potential (mV)	30.97
Primary particle radius (nm)	27.0
Ionic strength (mM)	1.0
Temperature (°C)	25.0

a Zhang, et al., 2010.

### Transdermal penetration

Porcine skin was cut into circular sections, 3 cm in diameter, positioned over a 40 ml EPA vial filled with 0.9% saline and held in place with a cap modified to hold the skin sample. The vial was filled such that the skin was in contact with the physiological saline and thus partially hydrated and isotonic. For both the light and dark trials, a dose of 200 µl of 1000 mg l^−1^ TiO_2_ was applied directly to the stratum corneum for the thin section and the permeate experiments, while the dose was applied every 30 min for the skin samples analyzed via ICP and the tape stripping experiments. Control experiments were conducted under dark conditions, i.e., no light and in an oven (Yamato DK-3, Japan) to ensure isothermal conditions. Like the light-exposed samples, the control experiments were conducted at 25°C. Although some signs of drying were observed in sunlight exposed skin samples, sunlight exposure to skin is a common environmental condition. Nevertheless, control experiments were conducted to ascertain the influence of potential sunlight damage to skin. The porcine skin was first exposed to sunlight for 180 min, with 200 µl of Nanopure water applied every 30 min and then subjected to the aforementioned TiO_2_ dosing regiment.

After exposure, the skin samples were rinsed several times with NanoPure water and subsequently with 5% HNO_3_. The permeate was also collected and digested for ICP elemental analysis, to determine the mass of titanium within the skin. To confirm transport into and through the dermis, the skin samples were embedded in resin and the top ∼500 µm were removed before ultramicrotomy was used to prepare 60 nm thin sections. Since the stratum corneum comprises the uppermost 10–40 µm, the thin sections were well within the dermis.

Triplicate skin samples used for sectioning via tape stripping were exposed to one 200 µL dose of 1000 mg L^−1^ TiO_2_ and either light or dark conditions for 90 min. After exposure, one tape strip was firmly adhered to the skin and then pulled from the skin following established methods [21], [22], [26], removing corneocytes and TiO_2_. Tape stripping was repeated, one strip at a time, for 40 times, yielding 40 tape strips. The absorbance was measured at UV_254_ to determine TiO_2_ content and Vis_430_ used to monitor the amount of corneocytes removed (Shimadzu Biospec 1601, Japan). The tape (Model 371, 3M, USA) was cut to 1.5×3.5 cm strips and adhered directly onto the cuvette holder in the spectrophotometer. Experiments were conducted in triplicate to produce samples for the ICP analysis

### Tissue embedding, sectioning and microscopy

At the conclusion of an experiment the tissues were initially rinsed several times with NanoPure water and 5% HNO_3_. Several skin samples were cut to yield roughly 1 mm by 1 mm by 10 mm vertical skin profiles. The embedding procedure is described in detail in the Supporting Information S1. A Leica 2065 ultramicrotome, equipped with automatic rotary sample advancement, was used to cut 60 nm thin sections from the skin sections. The thin sections were placed on TEM grids and analyzed on an STEM stage with an FEI - Nano600 SEM with a STEM attachment using an EDAX energy dispersive X-ray spectrometer.

## Results and Discussion

Photons appear to provide sufficient energy to dislodge loosely bound nanoparticles or small clusters of nanoparticles from the secondary minimum and induce partial nanoparticle disaggregation. To evaluate this hypothesis, the force-separation distance profile for two 27 nm TiO_2_ nanoparticles was calculated using DLVO theory [27], [28]. The calculation indicates that 3.9×10^−21^ J are needed to release a particle bound in the secondary minimum. When irradiated with UVA light, each primary particle can absorb up to 1.4×10^−15^ J s^−1^, which is much greater than the energy required to overcome the secondary minimum energy (calculation details in Supplementary Information). Given the energy requirement needed to disaggregate particles form the secondary minimum, even infrared light provides enough energy to release NPs from the secondary minimum. Since photons below the band-gap energy can induce disaggregation, it is unlikely that the UV conversion of TiO_2_ to a hydrophilic state, with trapped surface charges, is responsible for the observed photodisaggregation phenomenon [29]. In addition to thermally induced molecular vibrations, these NPs are infrared active; direct absorption of IR photons can induce vibrational modes. [30], [31]. The photons thus provide more than sufficient thermal energy to induce disaggregation of NPs, which can diffuse away from the secondary minimum. However, the nanoparticles in the core of the NP clusters are held at the primary minimum, which explains why the NP clusters are not fully disaggregated. Once the NPs dissipate the excess thermal energy, they can be recaptured in the secondary minimum.

### Photoinduced disaggregation

Real-time measurements of the hydrodynamic diameter during irradiation of TiO_2_ NPs in deionized (DI) water with a Xenon (Xe) lamp shows rapid disaggregation from 282.9 nm (7.3 std. error) before light to 246.2 nm (2.7 std. error), based on the average of the first 4 measurements after 10 min irradiation with light (Figure 1). The average hydrodynamic diameter after light exposure, also computed as the average of four measurements, was 230.6 nm (std. error 2.5). Although there is measurement scattering, as is typical with DLS, the differences in treatments are statistically significant; comparing the ‘before light’ and ‘light’ treatment sizes yields a 1.1×10^−5^ p-value, a p-value of 2.7×10^−24^ for the ‘light’ and ‘after light’ treatments and a p-value of 0.006 for the ‘before light’ and ‘after light’ treatments. The mean primary TiO_2_ NP diameter is 27 nm, indicating that the initial aggregates are clusters of dozens of NPs, as has been observed by other researchers [9], [11]. The initial rate of disaggregation is rapid but quickly decelerates; aggregate size increases again upon extinction of the light source (after 180 min in Fig. 1). Prolonged light exposure does not lead to full disaggregation since the core cluster of nanoparticles are bound by solid-state necks [13]. We found that light effectively disaggregates the agglomerated particles held by weak DLVO forces, but not those particles held by irreversible chemical bonds. Analysis of the first 10 min. of disaggregation or re-aggregation shown in Figure 1 indicates that the rates of disaggregation (−2.27 min^−1^), and re-aggregation (2.01 min^−1^) are similar; these kinetics suggest that the dislodged nanoparticles migrate away from the core and back at a rate, seemingly controlled by Brownian motion.

As an independent confirmation of DLS, we tracked disaggregation with 100 nm pore size filters. To perform the experiments we dispensed TiO_2_ NPs onto the filter, irradiated the dispersion, and analyzed the permeate for titanium. Since the pore size of the membrane (100 nm) is much smaller than the hydrodynamic diameter of the dispersed NPs (∼270 nm) the observation of titanium in the permeate confirms the influence of light on aggregation state. We found that after 120 and 180 min of exposure, 0.006% and 0.025% of the total TiO_2_ dose applied was found in the permeate. This corresponds to Ti concentrations in the 120 and 180 min treatments of 0.67 mg L^−1^ (std. error 0.04) and 2.9 mg L^−1^ (std. error 0.48) and approximately 3×10^11^ and 1×10^12^ particles, respectively, considering the mass of primary TiO_2_ nanoparticles for the calculation. Permeate from control experiments conducted at 25°C in the dark had only traces of titanium near the detection limit (ca. 5 ppb) after 300 minutes. We also conducted similar experiments with 10 nm filters and did not detect any titanium in the permeate, which was expected for 27 nm primary particles; ICP analysis confirmed that the there was no Ti in the permeate. As a percentage, the quantity of TiO_2_ that penetrated the filters was low since only a few primary particles were dislodged from the secondary minima of the aggregates. However, the importance of this experiment was to show that not only can small aggregates or primary particles pass through small pores, but also that light induces the transport of NPs through the filter.

**Figure 2:**
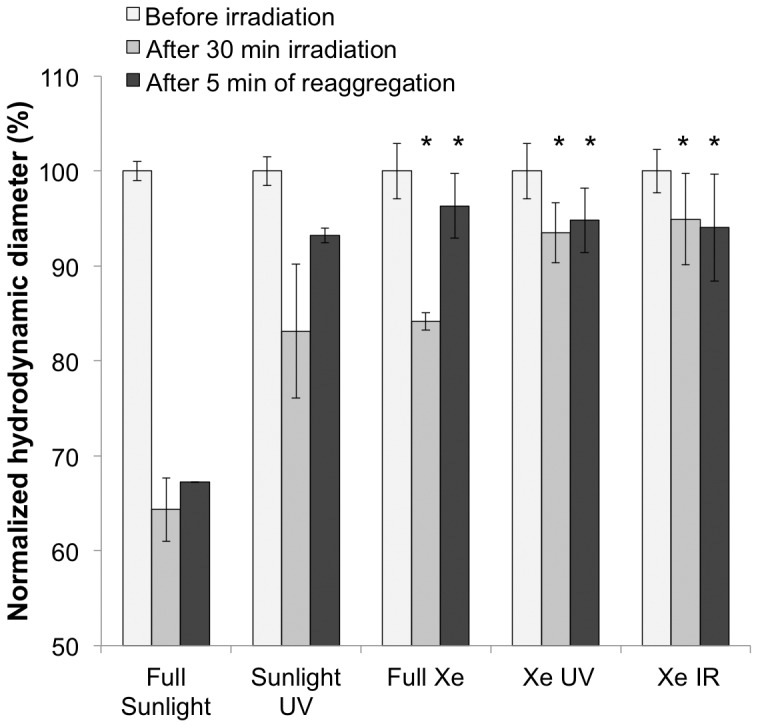
Induced disaggregation after irradiation of 100 mg L^−1^ TiO_2_ NP suspension with full spectrum sunlight and Xe arc lamp, as well as UVA or IR filtered light. In general the exposures with the greatest intensities induced the largest disaggregation. Irradiation was conducted for 30 min. for all experiments. In general a degree of reaggregation was observed after a few minutes. Error bars reflect a standard deviation. Stars indicate statistical significance with a p-value≤0.05.

Photoinduced disaggregation was more pronounced under natural sunlight (Figure 2). The initial aggregate hydrodynamic diameter before exposure to sunlight was 253 nm, which decreased to 159 nm (37%) after 30 min. After a few minutes in the dark the NP clusters began to reaggregate to the secondary minima. The data reflected in Figure 2 were from samples measured using the Malvern Zetasizer almost immediately after exposure but not in real time, as was done for the results in Figure 1. Sunlight intensity (ca. 500 W m^−2^) was greater than the Xe lamp (300 W m^−2^) and marked differences in photoinduced disaggregation were observed. Using bandpass filters (UG1, KG3, RG 850, Schott, USA), disaggregation of TiO_2_ can be accomplished with lower energy infrared (IR) photons up to the more energetic ultraviolet (UV). For the full spectrum Xe and Xe UV experiments, the decrease was 16% and 6% immediately after irradiation. To investigate the possible influence of surface charge accumulation and UV-induced hydrophilicity as a repulsive force, the ζ-potential was measured before and after irradiation. The ζ-potential decreased 5.6 mV, from the initial 39.0±0.4 mV. This ζ-potential decrease is insufficient to substantially modify the balance of Derjaguin-Landau-Verwey-Overbeek (DLVO) forces [32].

The polydispersity index (PDI), a measure of distribution of particle sizes in the system, was found to increase after light exposure (see Table S1). The increase in average PDI after each exposure indicates that there are more particles of different sizes present in the system and therefore provides evidence that irradiation results in the release of a few nanoparticles or small clusters from the aggregate. We also monitored the volume distribution and intensity (Figure S3), which corroborate the observed phenomenon.

### Transdermal penetration of TiO_2_ nanoparticles

To evaluate the implications of photoinduced disaggregation, we applied an aqueous dispersion of TiO_2_ NPs to isolated porcine skin sections and irradiated the samples. Although not identical to humans, pig skin is often used as a close analogue for percutaneous absorption and transdermal drug delivery studies [33]. In pigs, epidermal thickness varies depending on location and ranges from 46 um on the abdomen to 59 um on the leg [33]. Mammalian corneocytes are typically spaced 100 nm apart with the intercellular spaces containing lipids, proteins and other macromolecules [34]. Given the intercellular distance, many nanoparticles may be small enough to pass through the intercellular spaces as suggested by our experiments which showed photoinduced disaggregation enables NP penetration through the 100 nm pores of a synthetic membrane.

**Figure 3:**
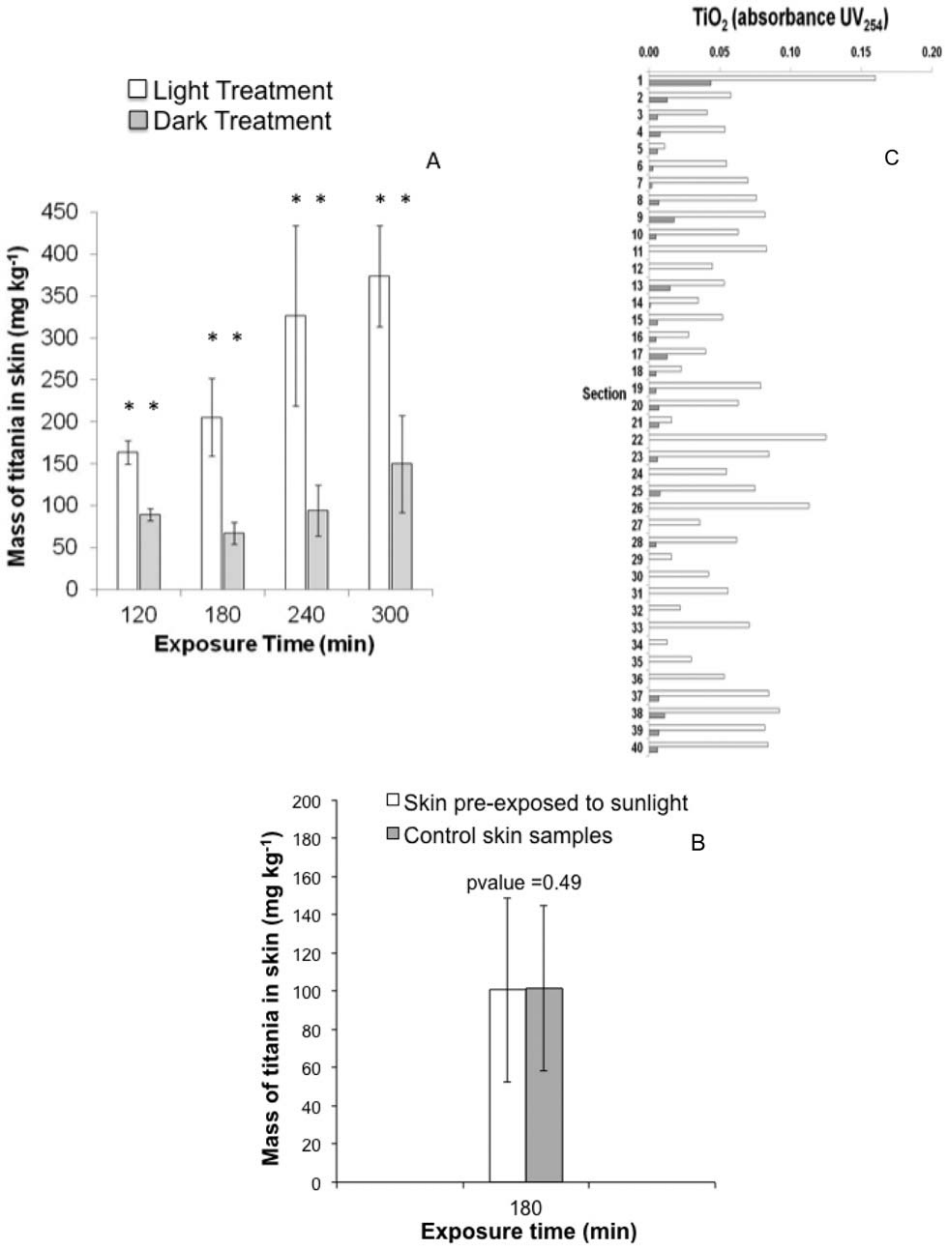
Mass of titanium in sunlight exposed skin grafts increasing with exposure to sunlight, indicating higher accumulation of TiO_2_ NPs within the skin (Panel A). The mass of titanium in the dark treatments is much lower compared to the mass found in the irradiated treatment, and does not increase significantly with time. Panel B: Skin pre-exposed to 180 min of sunlight and then subjected to 180 min of sunlight and TiO_2_ doesn't accumulate more TiO_2_ than skin not pre-exposed to sunlight. Control samples in Panel B were skin samples not pre-exposed to sunlight but subjected to the TiO_2_ dosing. Skin sections show that little to no titanium is found in the dark treatment, while it is found in all 40 sections for the irradiated sample (panel C). Stars indicate statistical significance with a p-value≤0.05. Molar exctinction coefficient was 2,169 cm^−1^ M^−1^.

Sunlight facilitated the disaggregation of NPs, which led to increased penetration of nanoparticles into and through the entire dermal profile (Figure 3A). Control experiments using skin pre-exposed to sunlight showed that potential sunlight damage to the skin did not enable or increase NP penetration (Figure 3B). Inductively coupled plasma (ICP) analysis of the full dermal profile showed that irradiated TiO_2_ was able to penetrate the skin and yielded concentrations as high as 370 mg TiO_2_ kg^−1^ in the skin profile after a 5 hr. sunlight exposure. The amount of irradiated titanium found within the skin increased with increasing exposure time (Figure 3A). By contrast, only 130 mg TiO_2_ kg^−1^ were present in unirradiated samples, and did not increase with time. Other studies have shown that a simple soap wash is suitable for removing TiO_2_ NPs from the surface of pig skin [24]. Since our washing method is more robust, with multiple washes of the skin surface with Nanopure water and 5% HNO_3_, we expect to have washed off almost all the TiO_2_ from the skin surface. Hence, these ICP results reflect titanium concentrations inside the skin. Since 5% HNO_3_ cannot dissolve TiO_2_ NPs, it is unlikely to increase their dermal transport, or that of dissolved titanium.

The permeate through the skin was collected from several samples to determine the mass of TiO_2_ that travelled through the entire dermal profile. After a 90 min exposure, the mass of titanium that permeated through the irradiated skin was 0.324 µg or 0.1% of the applied amount. In a similar experiment using 0.22 um filters as a membrane and an equivalent dose of titanium and exposure time, 1.2% of titanium passed through the filters. The small amount of TiO_2_ found in the permeate represents a small fraction of the total dose, since only a small fraction of the NPs can disaggregate and migrate through the skin. Most of the TiO_2_ applied remains in the large clusters.

Thin skin sections also showed increased titanium within the skin of irradiated samples. After 90 min exposure to light, TiO_2_ NPs were observed in detectable amounts on 40 successive sections (Figure 3C, see also Figure S4). In unirradiated samples, there was either no TiO_2_ or the signal was very small after the first section. This provides evidence of transdermal penetration facilitated by photo-induced disaggregation.

**Figure 4:**
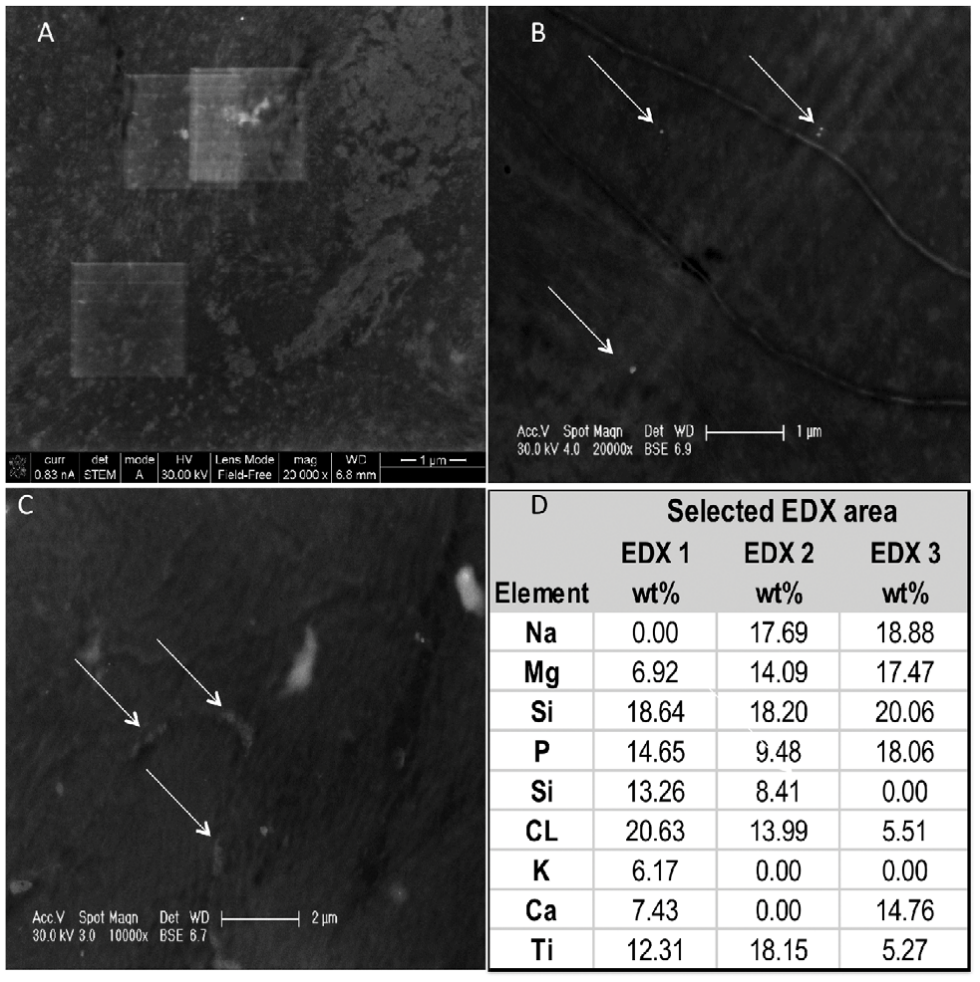
STEM micrographs of TiO_2_ penetration into dermal tissue (Panels A–C). EDX analysis confirms the bright spots imaged in the skin tissue contain titanium, with as much as 18.15 weight (wt) % (Panel D). Note the small TiO_2_ aggregates and primary particles in the vasculature of the dermis (B). Additionally, tens to hundreds of NPs are found well within the dermis (C). All images are from sections 500 µm below the skin surface.

Scanning transmission electron microscope (STEM) analysis of several thin sections (Figure 4) showed tens to hundreds of primary NPs or small clusters in the interstitial spaces of the porcine dermis after irradiation. Energy dispersive X-ray (EDX) confirmed that these small particles contained titanium and were present in significant amounts even at depths up to 500 µm below the skin surface. STEM and EDX analyses of the control samples, *i.e.*, dark conditions, showed that TiO_2_ was only found on the surface of the stratum corneum (images not shown).

## Conclusions

The ability of light to facilitate NP transport in the environment and through dermal tissue has health and environmental implications. The results indicate that TiO_2_ nanoparticle clusters can be disaggregated by natural and artificial light, and that the effect is related to light intensity. Photo-induced disaggregation is not limited to UV light. The dislodged nanoparticles and small clusters can then penetrate deep into the skin. Corneocytes are spaced 100 nm in most mammals and thus our work with 100 nm pore size filters that showed light facilitates increased NP penetration provides greater evidence that dermal penetration is influenced by photoinduced disaggregation. To the best of our knowledge, there have been no studies that investigated trans-dermal mobility of NPs in the presence of light. As is common in dermal penetration studies, this study used pig skin in lieu of human skin. Additionally, our work relied on pig skin samples that were not subjected to the mechanical forces that have been shown to increase the mobility of NPs through skin [21]. The stratum corneum, the outer layer of corneocytes responsible for retaining many NPs, is generally two times thicker in pigs than in humans, which suggests that NP penetration of human skin may be more significant. The ability of light to disaggregate NPs also has potential application in industries which have a need to manipulate aggregate size in aqueous media, e.g., pharmaceutics, paint, metallurgy, rubber manufacturing.

## Supporting Information

Supporting Information S1 The calculation protocol needed to estimate the amount of light energy absorbed per particle is included in Supporting Information S1, as well as the theory behind our DLVO calculations and the parameters used to make our DLVO calculations. Additional DLS data, size as volume and intensity, are also included. We have also included additional TiO_2_ penetration data. Finally, TEM images of the TiO_2_ materials used for this work appear in SI.
(DOCX)

Figure S1 Transmission electron micrographs of TiO_2_ used for our work. Micrograph courtesy of Ivy Ji at UCLA.
(TIFF)

Figure S2 Spectrum of the Xenon arc lamp.
(TIFF)

Figure S3 Representative volume and intensity distribution results for a 100 mg L-1 TiO_2_ solution before and after irradiation. A shift in volume distribution of smaller particles is clearly present after 30 min irradiation. Similarly, the intensity of smaller particles also increases after irradiation.
(TIFF)

Figure S4 The mass (absorbance) of corneocytes removed with each section via tape stripping from both the light and dark exposed skin grafts (Panel A). Panel B presents the amount TiO_2_ found per section normalized by the mass of corneocytes removed with each section.
(TIFF)

Table S1 The polydispersity index (PDI) results for sunlight experiments are presented in Table S1 for unirradiated and irradiated 100 mg L^−1^ TiO_2_ samples. The samples were irradiated by the UVA fraction of natural sunlight for 30 min.
(TIFF)
