# Supplementary material for: Photoinduced Disaggregation of TiO2 Nanoparticles Enables Transdermal Penetration
Source: PLoS One. 2012 Nov 14;7(11):e48719. doi: 10.1371/journal.pone.0048719 (PMC3498245; doi:10.1371/journal.pone.0048719)
Supplement: Supporting Information S1 — The calculation protocol needed to estimate the amount of light energy absorbed per particle is included in Supporting Information S1, as well as the theory behind our DLVO calculations and the parameters used to make our DLVO calculations. Additional DLS data, size as volume and intensity, are also included. We have also included additional TiO2 penetration data. Finally, TEM images of the TiO2 materials used for this work appear in SI. (DOCX) [file pone.0048719.s001.docx]

**Photoinduced Disaggregation of TiO_2_ Nanoparticles Enables Transdermal Penetration**

Samuel W. Bennett, Dongxu Zhou, Randall Mielke & Arturo A. Keller^◊^

University of California Center on the Environmental Implications of Nanotechnology and Bren School of Environmental Science and Management, University of California at Santa Barbara, California, United States of America

^◊^ **Corresponding author** E-mail: [keller@bren.ucsb.edu](mailto:keller@bren.ucsb.edu); Phone: 805.893.7548

**Supplementary information**

**Particle energy absorption calculation**

To estimate the amount of light energy absorbed by each particle we first calculate the number of TiO_2_ molecules per particle. Using a density of 4 g cm^-3^ the number of molecules is 310,728 TiO_2_ per 27 nm particle. Next the total number of molecules in the system was calculated. We assumed a monodisperse sample and divided the ratio of total TiO_2_ molecules in the system and TiO_2_ molecules per particle by the intensity of light received by the 1 cm^2^ cuvet, assuming 100 mg L^-1^ TiO_2_ absorbs 100% of UVA light. Even if the amount adsorbed is smaller, the energy available is more than sufficient to dislodge a TiO_2_ nanoparticle from the secondary minimum.

**DLVO Calculations**

*DLVO theory calculation.* Two interparticle interactions are considered in classic DLVO theory, electrostatic repulsion and van der Waals attraction. Assuming low surface charge, spherical particles of equal size, thin electric double layers compared to the particle size, the electrostatic repulsion, *F_R_*, is given by (Elimelech et al., 1995),


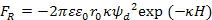


Where *ε* is the permittivity, *r_0_* is the particle size, *ψ_d_* is the particle surface potential, and *H* is the separation distance between two particles. The inverse Debye length, *κ*, can be obtained by,

Where *ze* is the ion charge, *N_A_* is Avogadro’s constant, *c* is ion concentration, and *k* is Boltzmann’s constant and *T* is the temperature. Under the assumptions that particles are spherical and of equal size, and the separation is small compared to particle size, the van der Waals attraction force can be calculated by the following equation,

$$F_{A}=\frac{Ar_{0}}{12H^{2}}$$

Where *A* is the Hamaker constant. Then the total energy is simply the summation of the two interactions.

$$F_{T}=F_{A}+F_{R}$$

**Tissue embedding for microscopy.**

The embedding procedure is as follows:

1. Rinse samples 3 times in NanoPure water
2. Place samples in glass vial and cover with 2% OsO_4_, for one hour
3. Rinse samples 3 times in NanoPure water
4. Dehydrate samples by covering for 20 min in solvent and then pour the solvent off; the samples are then soaked in 25% ethanol, followed by 50%, ethanol, 75% ethanol, 100% ethanol twice, 50-50% ethanol-acetone, and finally 100% acetone.
5. Following dehydration, the samples are covered in 50% acetone and 50% resin and stored at room temperature for 12 hr.
6. Samples are then placed in TEM block and covered with resin.
7. The TEM block is placed in an oven at 60º C for 24 hr.
8. The resin embedded samples are removed from the block and prepared for ultramicrotomy.
