# Supplementary figures and images for: Photoinduced Disaggregation of TiO2 Nanoparticles Enables Transdermal Penetration
Source: PLoS One. 2012 Nov 14;7(11):e48719. doi: 10.1371/journal.pone.0048719 (PMC3498245; doi:10.1371/journal.pone.0048719)

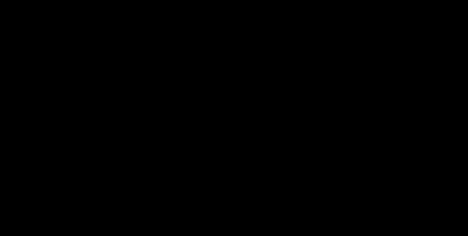

Supplement: Figure S1 — Transmission electron micrographs of TiO2 used for our work. Micrograph courtesy of Ivy Ji at UCLA. (TIFF) [file pone.0048719.s002.tif]

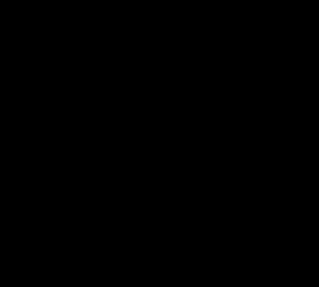

Supplement: Figure S2 — Spectrum of the Xenon arc lamp. (TIFF) [file pone.0048719.s003.tif]

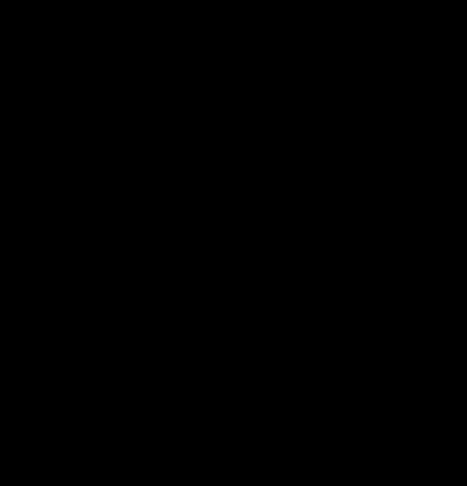

Supplement: Figure S3 — Representative volume and intensity distribution results for a 100 mg L-1 TiO2 solution before and after irradiation. A shift in volume distribution of smaller particles is clearly present after 30 min irradiation. Similarly, the intensity of smaller particles also increases after irradiation. (TIFF) [file pone.0048719.s004.tif]

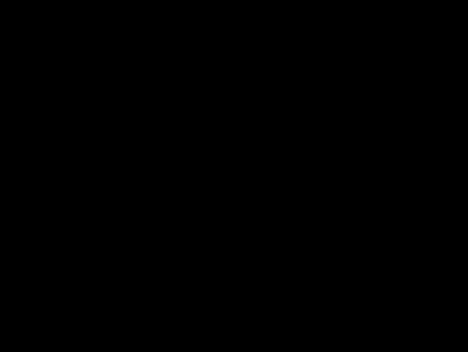

Supplement: Figure S4 — The mass (absorbance) of corneocytes removed with each section via tape stripping from both the light and dark exposed skin grafts (Panel A). Panel B presents the amount TiO2 found per section normalized by the mass of corneocytes removed with each section. (TIFF) [file pone.0048719.s005.tif]

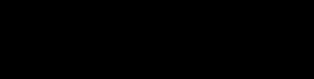

Supplement: Table S1 — The polydispersity index (PDI) results for sunlight experiments are presented in Table S1 for unirradiated and irradiated 100 mg L−1 TiO2 samples. The samples were irradiated by the UVA fraction of natural sunlight for 30 min. (TIFF) [file pone.0048719.s006.tif]
